# Bilateral Asymmetrical Fracture Dislocation of Shoulder with Rare Combination of Injuries after Epileptic Seizure: A Case Report

**DOI:** 10.5704/MOJ.1703.011

**Published:** 2017-03

**Authors:** A Sharma, S Jindal, MS Narula, S Garg, A Sethi

**Affiliations:** Department of Orthopaedics, Government Multispecialty Hospital, Chandigarh, India

**Keywords:** bilateral, shoulder, dislocation, fractures, seizures

## Abstract

The incidence of bilateral gleno-humeral joint dislocation is rare, is almost always posterior and is usually caused by sports injuries, epileptic seizures, electrical shock, or electroconvulsive therapy. Bilateral fracture-dislocation is even rarer, with a few cases reported in the literature. We report an unusual case with dislocation of the both glenohumeral joints in opposite direction after a seizure episode, with fracture of greater tuberosity on one side and of the lesser tuberosity on the contralateral side. Although there have been a few reports of bilateral asymmetric fracture dislocations of the shoulder in the past, an injury pattern resembling our case has, to the best of our knowledge, not been described in the literature so far. This report includes a detailed discussion regarding the mechanism of injury in a case of asymmetrical dislocation following a seizure episode. At final follow-up, the patient had healed fractures, painless near normal range of motion with no redislocations.

## Introduction

The shoulder, by virtue of its anatomy and biomechanics, is one of the most unstable and frequently dislocated joints in the body, accounting for nearly 50% of all dislocations. On the contrary, the incidence of bilateral gleno-humeral joint dislocation is rare, is almost always bilateral, posterior and is usually caused by sports injuries, epileptic seizures, electrical shock, or electroconvulsive therapy^1^. Bilateral fracture-dislocation is even rarer, with very few cases reported in the literature^2^. We report an unusual case with dislocation of the both gleno-humeral joints in opposite directions with fracture of greater tuberosity on one side and of the lesser tuberosity on the contralateral side.

## Case Report

A 40 years old female reported to the emergency department of a level II trauma center after an epileptic convulsion with complaints of pain and restriction of movements of both shoulder joints. Physical examination revealed hollowness below the acromion process on either side with flattening of the normal contour of the shoulders with the arm held in external rotation and abduction on the left side and in slight internal rotation on the right side. No peripheral neurological deficit was noted. No specific history of the mode of injury could be elicited as there was no witness of the episode and the patient could not clearly recall the event.

**Fig. 1:**
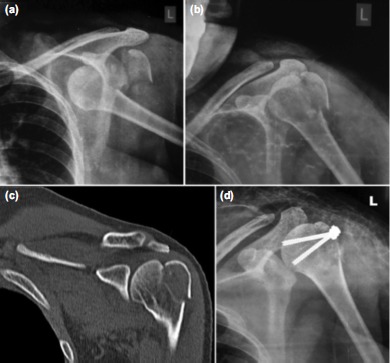
(a) Initial radiograph, (b) Post reduction radiograph, (c) Pre-op CT scan coronal section and (d) Post-op radiograph of left shoulder.

**Fig. 2:**
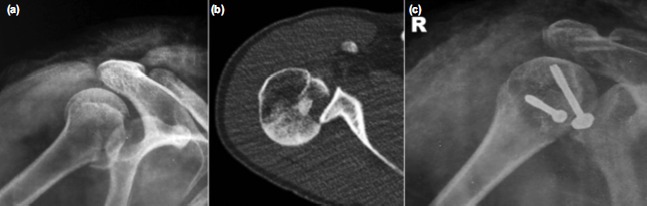
(a) Pre-op radiograph, (b) Pre-op scan axial section and (c) Post-op radiograph of right shoulder.

Radiographs revealed anterior shoulder dislocation with a displaced greater tuberosity fracture on the left side (Fig. 1) and posterior dislocation with lesser tuberosity fracture on the right side (Fig. 2). Both the dislocations were promptly reduced by closed manipulation in the emergency department and post-reduction radiographs showed persistent but decreased displacement of both the tuberosities (more on the side with the greater tuberosity fracture) (Fig. 1a,1b, 2a). A CT scan of both shoulder joints was done for better demonstration of the fracture pattern (Fig. 1c, 2b).

After preoperative workup, anesthetic evaluation, physician consultation and informed consent, patient was taken up for surgery. Lesser tuberosity fracture on the right side was fixed using two partially threaded cannulated screws using deltopectoral approach to the shoulder (Fig. 2c). The greater tuberosity fracture on the left side was similarly fixed using two partially threaded cannulated screws via direct lateral approach (Fig. 1d). Post-operatively both the shoulders were immobilized in a shoulder immobilizer for three weeks. Physical therapy was started as tolerated by the patient at four weeks. She was able to resume her daily activities by two months. At one year follow-up, the patient was free of pain in both shoulder joints and radiographs revealed consolidation at the fracture site with no re-dislocations occurring during the follow up period. After aggressive physiotherapy, the range of motion was near normal on the left side but slight limitation of abduction (range 0-120 degrees) was noted on the right side.

## Discussion

The unique aspect of this case is the simultaneous bilateral asymmetric opposite displacement of the dislocation of the shoulders with greater tuberosity fracture on one side (associated with anterior dislocation) and lesser tuberosity fracture on the opposite side (associated with posterior dislocation of glenohumeral joint). Although there have been a few reports of bilateral asymmetric fracture dislocation of the shoulder in the past^3^, an injury pattern resembling our case has not been described in the literature, to the best of our knowledge.

Bilateral dislocations of the shoulder are a rare entity and are usually caused by sports injuries, epileptic seizures, electrical shock, or electroconvulsive therapy, hypoglycemic episodes or dyskinetic disorders. They almost always occur in the same direction and often posteriorly directed, especially when associated with seizures. The biophysical basis of this posteriorly directed dislocation is believed to be violent involuntary contraction of muscles around the shoulder, especially the internal rotators and adductors, which are stronger in comparison to the external rotators and abductors. The resultant sustained pull and direction of shoulder joint causes it to dislocate posteriorly^4^. Nevertheless, there have been reports of anterior shoulder dislocations after involuntary muscle contractions. In cases with anterior dislocation after a seizure episode, most authors believe that the mechanism was due to fall in an unconscious state with the flail upper limb held in abduction and extension at the shoulder, causing impingement of the greater tuberosity on the acromion process, levering the humeral head out of glenoid. Further, the humeral head is pushed downward by the rotator cuff, which is finally displaced anteriorly by the flexors and external rotators. Whether the anterior dislocation in a case of involuntary muscle contractions as in. seizures is caused by trauma after fall or due to muscular forces still needs to be evaluated as there have been reports in which forceful muscular contractions without any history of fall have led to anterior dislocation^5^. Thus, in any case of shoulder dislocation after involuntary muscular spasm, a specific history of fall and mechanism of injury must be sought to ascertain the cause of injury. Moreover, there is a need for a detailed study of such cases to define the actual mechanism of anterior dislocation.

In the rare case of an asymmetrical bilateral dislocation, attention may be distracted to the more evident lesion, which is the anterior dislocation. This may lead to delay in diagnosis especially. in an unconscious patient in a post-ictal state.

The greater tuberosity is displaced in approximately 15% of all anterior dislocations. A displaced greater tuberosity fracture usually signifies an associated rotator cuff injury and if not reduced anatomically can lead to permanent disability and decreased range of motion. Fracture of the lesser tuberosity of the humerus often occurs concurrently with fracture of the proximal humerus or posterior dislocation of shoulder. Open reduction and internal fixation is recommended if the displacement is greater than 5 mm or angulation is more than 45° because displacement caused by the pull of subscapularis tendon may lead to aggravation, mal-union, impingement of the coracoid process, or dislocation of the tendon of the long head of biceps. In the present case, open reduction and internal fixation of both greater and lesser tuberosity fragments were performed using partially threaded cancellous cannulated screws. The patient achieved satisfactory outcome at follow up at 12 months.

There is no conflict of interest and no financial assistance was received for this study and report.
